# Rainfall patterns during barley seed development underlie genomic variation for germination after flooding

**DOI:** 10.1093/plphys/kiaf563

**Published:** 2025-11-09

**Authors:** Eva Maria Gómez-Álvarez, Margherita Marazzini, Leonardo Caproni, Luca Magnani, F Cardarelli, Matteo Dell’Acqua, Pierdomenico Perata, Chiara Pucciariello

**Affiliations:** Institute of Plant Sciences, Scuola Superiore Sant'Anna, 56010 Pisa, Italy; nanoPlant Center @NEST, Center of Plant Sciences, Scuola Superiore Sant’Anna, 56127 Pisa, Italy; NEST Laboratory, Scuola Normale Superiore, Piazza San Silvestro 12, 56127 Pisa, Italy; Institute of Plant Sciences, Scuola Superiore Sant'Anna, 56010 Pisa, Italy; Institute of Plant Sciences, Scuola Superiore Sant'Anna, 56010 Pisa, Italy; NEST Laboratory, Scuola Normale Superiore, Piazza San Silvestro 12, 56127 Pisa, Italy; Institute of Plant Sciences, Scuola Superiore Sant'Anna, 56010 Pisa, Italy; Institute of Plant Sciences, Scuola Superiore Sant'Anna, 56010 Pisa, Italy; Institute of Plant Sciences, Scuola Superiore Sant'Anna, 56010 Pisa, Italy; nanoPlant Center @NEST, Center of Plant Sciences, Scuola Superiore Sant’Anna, 56127 Pisa, Italy

## Abstract

The diversity of plant genetic resources is the result of complex evolutionary processes, including adaptation to environmental stresses. High precipitation levels during the growing season may result in soil flooding events that place major constraints on crop productivity. Barley (*Hordeum vulgare*) is one of the most important cereals worldwide and serves as a model for studying the molecular responses of plants to climate change, due to its wide adaptability and diffusion to different environments. We explored the genetic associations of a global collection of barley landraces and wild relatives with rainfall regimes recorded in their growing areas. We found that the rainfall patterns observed during the driest months of the year and corresponding to the seed development period correlated significantly with the subsequent capacity of barley accessions to germinate after flooding. We then conducted an environmental genome-wide association study (eGWAS) and analyzed exome sequencing data, which revealed a narrow region on barley chromosome 1 with a possible influence on barley response to rainfall patterns. Using molecular approaches, we identified gene candidates involved in seed morphology and dormancy that are crucial for barley germination in soil after a flooding event in a natural environment.

## Teaser

Barley's adaptation to rainfall patterns is associated with genetic variations that contribute to controlling seed germination after flooding.

## Introduction

Ensuring future food security depends on the ability of crops to adapt to different climates and to thrive on marginal lands. Over the last decades, breeding research has directed its scope to yield performance, focusing on the production of commercial varieties that incorporate useful traits for plant responses to the effects of climate change. Currently, locally adapted plant genetic resources, especially crop wild relatives and landraces, are of primary interest, since they are reservoirs of extensive and untapped genetic diversity for local and extreme adaptation.

The characterisation of crop genetic diversity is crucial for the identification of beneficial alleles for genetic improvement (Bohra et al. 2022). While many *ex situ* collections have been assembled and are available to the scientific community, the diversity of crop wild relatives and landraces is still not comprehensively represented. The identification and conservation of these materials is crucial, given the changes in spatial distributions of plant species and because of climate change (Castañeda-Álvarez et al. 2016).

In germplasm collections, wild and landrace accessions can be traced back to their geographical sites of original collection, using passport data, which provide valuable information about the pedoclimatic diversity of their growing area (Canella et al. 2022). This information can be used to test associations between local diversity and genomic loci responsible for adaptation via environmental genome-wide association studies (eGWAS) (Brunazzi et al. 2018). These loci and their allelic diversity are a useful tool to direct plant breeding toward environmental adaptations (Mattila et al. 2016).

Previous studies looked into barley genetic resources in Ethiopia, identifying candidate genes associated with temperature and rainfall variation. These approaches have also highlighted areas where the current diversity of barley genetic resources may be poorly adapted to future climates (Abebe et al. 2015; Caproni et al. 2023).

In this work, we explored the precipitation data of a subgroup of barley landraces and wild accessions belonging to the WHEALBI collection (https://www.wheatinitiative.org/whealbi, last connection: 19/06/2025). The amount of rain is potentially predictive of flooding events occurring in certain regions and may reveal adaptive local responses in barley accessions.

We found that the amount of precipitation in the driest month and warmest quarter of the year significantly correlates with the germination phenotype after a flooding event. Using barley exome genotyping data, we carried out an eGWAS, which led to the identification of a genomic region on chromosome 1 harboring two significant Marker Trait Associations (MTAs). Within this region, we identified candidate genes in linkage with the signals that are involved in seed permeability and dormancy. These genes exhibit distinct alleles in barley accessions originating from environments with divergent rainfall regimes during the driest months. These accessions respond differently to prolonged soil flooding, exhibiting morphological, physiological and molecular adaptations.

Our hypothesis is that divergent rainfall regimes during barley seed development are associated with allelic variation at the locus influencing subsequent germination. This genetic variation contributes to differential responses to long flooding events under natural environmental conditions.

## Results and discussion

### Geographical diversity of barley landraces and wild accessions

**Figure 1.**
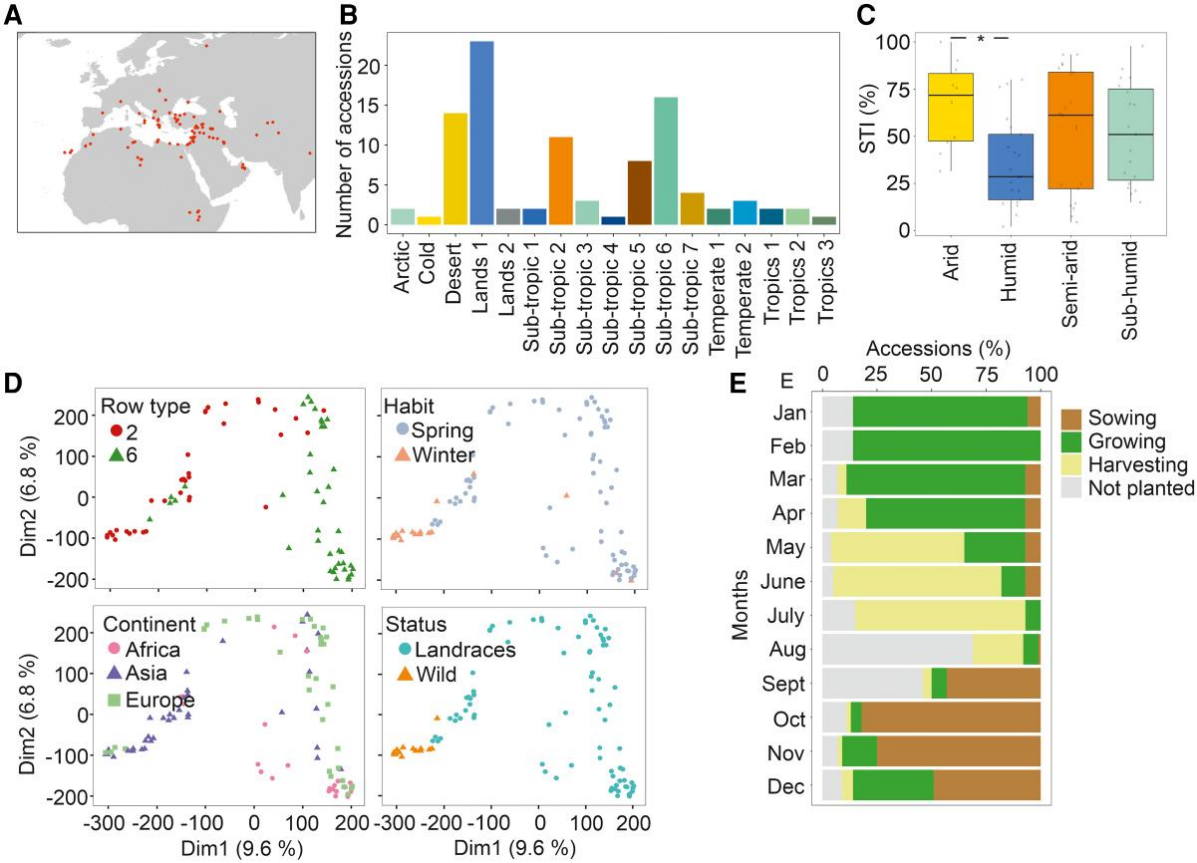
Geographic distribution, agroclimatic classification, and genetic structure of wild and landrace barley accessions from the WHEALBI collection. **A)** Map of the selected wild and landrace barley WHEALBI accessions. **B)** Number of accessions belonging to the different agroclimatic regions. Lands 1: lands with ample irrigated soils; Lands 2: lands with sever soil limitations; Sub-tropics 1: cool, semi-humid; Sub-tropics 2: cool, semi-arid; Sub-tropics 3: cool, sub-humid; Sub-tropics 4: moderately cool, humid; Sub-tropics 5: moderately cool, semi-arid; Sub-tropics 6: moderately cool, sub-humid; Sub-tropics 7: warm, semi-arid; Temperate 1: cool, moist; Temperate 2: cool, wet; Tropics 1: highland, humid; Tropics 2: highland, sub-humid; Tropics 3: lowland, sub-humid. **C)** Agroclimatic regions grouped based on the humidity of the environment in relation to the STI of the barley accessions. **D)** Principal Component Analysis (PCA) on the exome of barley accessions, visualizing the row type, growth habit, continent of origin, and biological status. Dim1, dimension 1; Dim2, dimension 2. **E)** Calendar of development of the barley accessions (FAO database). The timeframe of each developmental phase of the agricultural cycle includes several months. Within each box, center lines denote median values, box limits extend from the 25th to the 75th percentile, whiskers denote 1.5 × interquartile range, and dots denote outliers. ANOVA followed by Tukey's HSD test (*P*-value < 0.05).

The barley WHEALBI collection holds over 400 accessions, including landraces and wild relatives. In a previous work, we explored a subgroup of cultivated genotypes, typing the capacity of de-hulled seeds to germinate promptly after a short period of submergence, and identified a large variation in the response (Gómez-Álvarez et al. 2023). Compared with other cereals, barley is very sensitive to submergence (Arduini et al. 2016). In germination, this is due to the inability to degrade starch in the endosperm under O_2_ deprivation and to the hypoxia-dependent activation of secondary dormancy in some accessions, which precludes germination when water recedes (revised by Gómez-Álvarez and Pucciariello 2022). In this study, we identified landraces and wild barley accessions from the WHEALBI collection that were georeferenced, allowing the exploration of climatic diversity at their sites of origin. The 110 identified barley accessions originate from Europe, Africa and Asia (Fig. 1A), thus representing the barley trajectories of diffusion after domestication in the Fertile Crescent (Bustos-Korts et al. 2019). The diversity of the subgroup, which represents contrasting environments and dissimilar genetic backgrounds, supports the identification of molecular drivers for local adaptation, which may have a high potential for breeding (Muñoz-Amatriaín et al. 2014).

The agroecological characteristics (FAO, https://gaez.fao.org/pages/data-viewer) of the sampling points of the selected accessions were explored (Fig. 1B). Most of the accessions originated from areas with different water regimes, such as ample irrigated soils, sub-tropics and desert/arid lands. When the different areas were grouped according to the humidity of the environment, four different habitats were identified, i.e. arid, humid, semi-arid and sub-humid (Supplementary Table S1). This broad diversity may contain the presence of phenotypes related to different rainfall regimes.

In our previous work, barley varieties, landraces and wild accessions were classified based on the submergence tolerance index (STI), which represents the percentage of de-hulled seed germination after a short submergence in comparison to air (Gómez-Álvarez et al. 2023). When the STIs of the accessions of the four different habitats were compared, those that originated from humid areas showed significantly lower values (and thus a lower capacity to germinate after a short submergence event) than accessions belonging to arid regions (Fig. 1C). This was surprising, since accessions that germinate rapidly, and which have possibly adapted to flooding conditions would be expected in the most humid regions. In humid areas, the selection against preharvest sprouting may have occurred, ensuring that short periods of rainfall during the maturation phase do not cause substantial crop losses.

We studied the genomic diversity based on single-nucleotide polymorphism (SNP) markers on the barley exome (Bustos-Korts et al. 2019). The visual separation of the collection's genomic diversity through multidimensional scaling (MDS) correlated with several traits that are represented by different colors in four different graphs (Fig. 1D). A major separation was identified between spring and winter growth habits that corresponds to the distinction between landraces and wild accessions and represents 9.6% of diversity (Fig. 1D).

Previously, molecular differences during barley grain development were shown to be responsible for the phenotype occurring during the subsequent seed germination (Gómez-Álvarez et al. 2023). Taking this aspect into consideration, we identified the putative calendar of development and cultivation of the selected wild and landrace accessions (FAO, https://gaez.fao.org/pages/data-viewer). The period May–July corresponds to the harvesting time for most of the accessions examined, which includes the phase of grain development for both landraces and wild accessions (Fig. 1E).

### Climatic variables correlate with germination traits.

**Figure 2.**
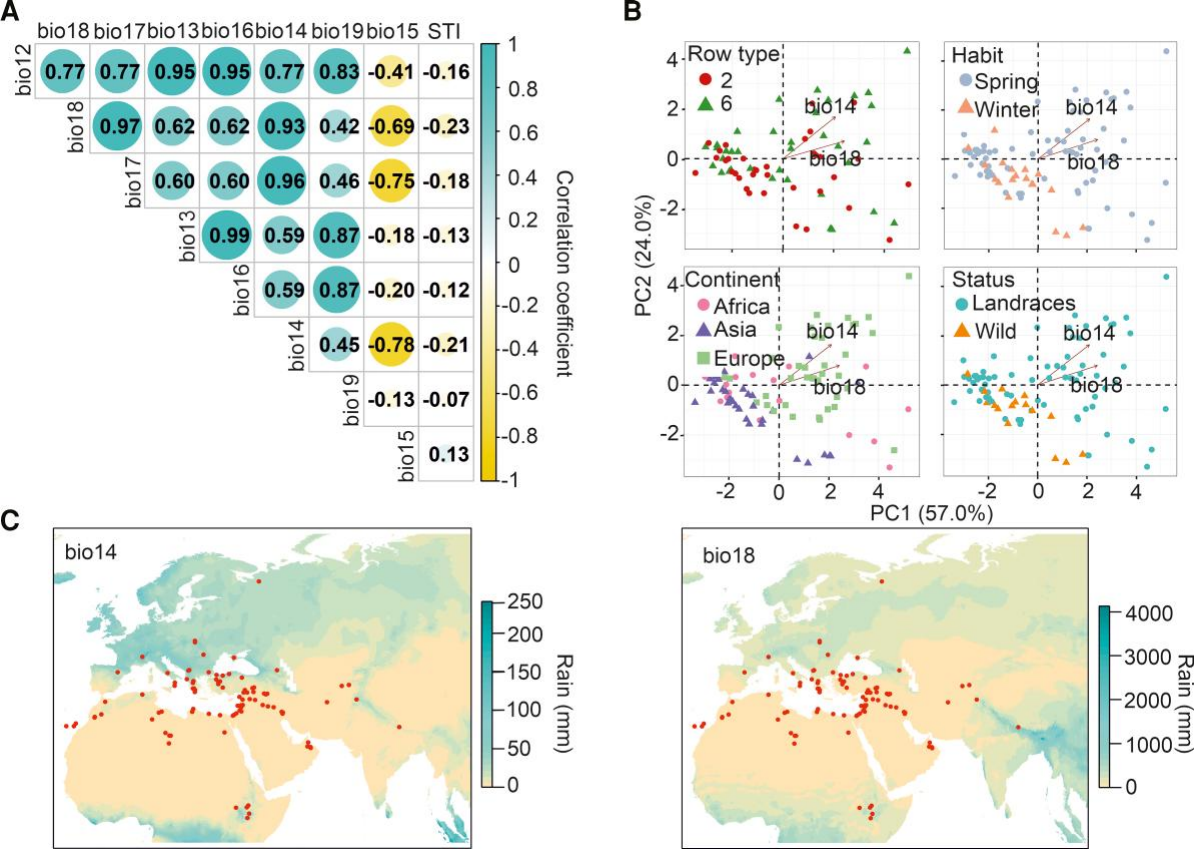
Correlation, principal component analysis (PCA), and geographic distribution of rainfall-related bioclimatic variables associated with barley tolerance. **A)** Correlation analysis of all the bioclimatic variables related to rainfall patterns (bio12-bio19, see Materials and Methods section) and STI. Spearman's rank correlation coefficient analysis, *P*-value < 0.05. For STI, only the correlations with bio14 and bio18 are significant. **B)** PCA of the bioclimatic variables considering the row type, growth habit, continent of origin, and biological status. Only bio14 and bio18 are plotted since they show a significant correlation with the STI. **C)** Rainfall pattern for bioclimatic variables bio14 and bio18 in the areas where the barley accessions were collected.

Eight bioclimatic variables related to rainfall patterns (bio12–bio19) obtained from the WorldClim2 database (https://www.worldclim.org/) were correlated with the STIs measured directly on the barley panel in our previous work (Gómez-Álvarez et al. 2023), which indicates the germination capacity of de-hulled seeds after a short submergence (Fig. 2A). The variable recording the amount of precipitation in the driest month (hereafter bio14), as well as the amount of precipitation in the warmest quarter of the year (hereafter bio18), correlated weakly, but significantly with the previously calculated STI (Spearman's rank correlation coefficient analysis −0.21 and −0.23, respectively, *P*-value < 0.05, Fig. 2A). The negative value of the correlation indicates that when the amount of precipitation in the driest month or in the warmest quarter of the year is low, the subsequent capacity to germinate after a short submergence event increases. These two bioclimatic variables are likely to represent the same period of the year and are significantly correlated (Spearman's rank correlation coefficient analysis 0.93, *P*-value < 0.05, Fig. 2A).

The bioclimatic variables were represented by a PCA, which revealed that bio14 and bio18 were positively associated with landraces from Europe, with a spring habit. The length of the arrows (bio14, bio18) represents the goodness of the association (Fig. 2B). For the selected accessions, bio14 and bio18 refer to the months from seed development to harvesting, i.e. May to July (Fig. 1E, Supplementary Table S1, http://www.fao.org/giews/en/). We studied bio14 and bio18 of the original collection site of the accessions, in order to visualize the rainfall distribution in those areas. Several barley accessions considered in this analysis originated from dry areas in summer (Fig. 2C, Supplementary Table S1).

In addition, a correlation analysis was performed of the monthly rainfall data for all the selected accessions (Supplementary Fig. S1). These results revealed that lower precipitation in July, the end of the harvesting season and one of the driest and warmest months of the year, significantly correlates with higher precipitation in November and December, when wild accession seeds may be available.

### Association analysis of marker—bioclimatic variables identifies a common overlapping region on chromosome 1

**Figure 3.**
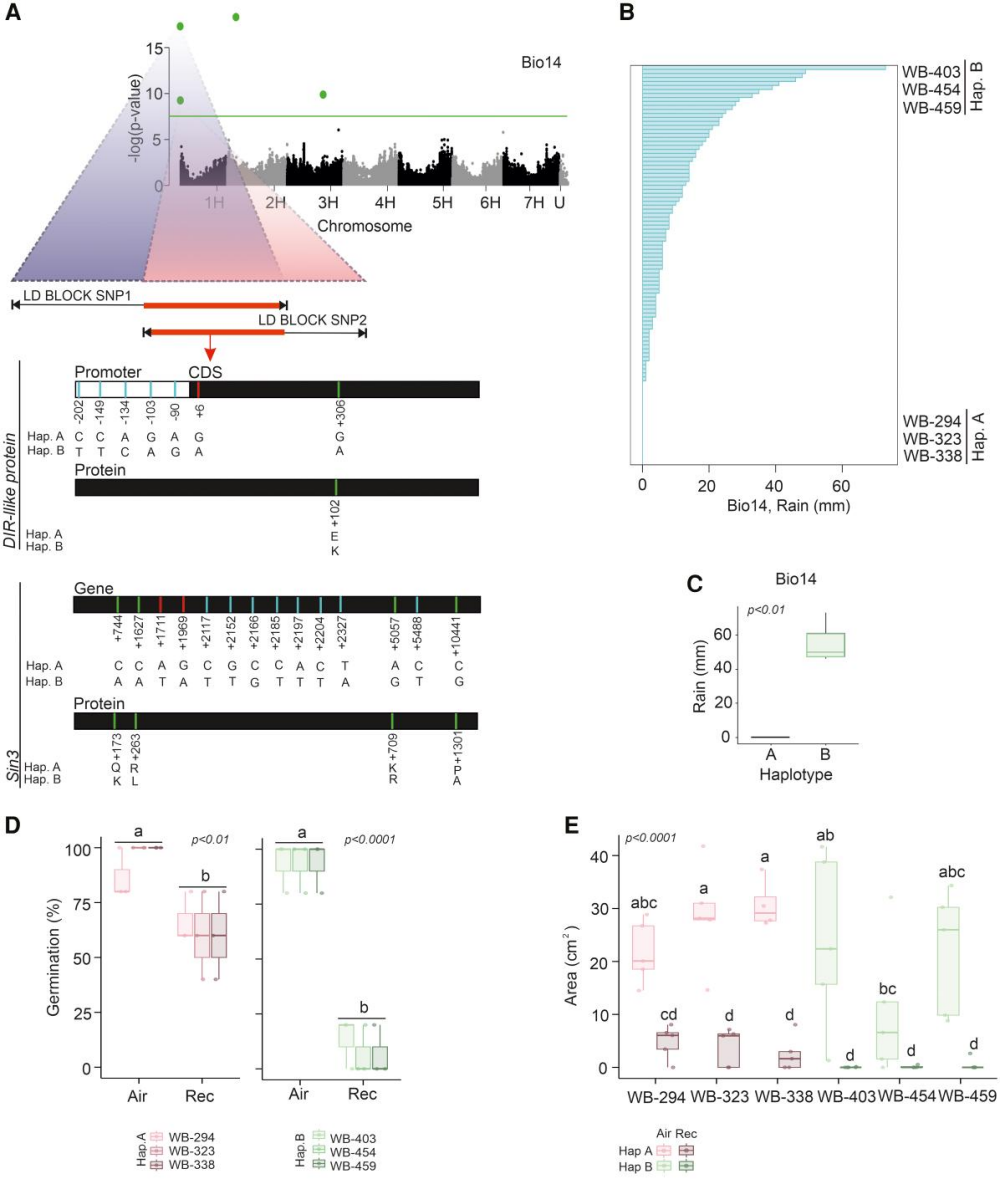
Environmental genome-wide association study (eGWAS) for rainfall bio14 variable and phenotype of extreme barley accessions. **A)** Manhattan plot of the eGWAS analysis of bio14 showing the haplotypes for the significant SNPs (FDR and Bonferroni, *P*-value < 0.05) and the LD blocks that show an overlapping region on chromosome 1. The line represents the Bonferroni threshold (α = 0.05). **B)** Bio14 distribution of the barley accessions. Haplotype A (WB-294, WB-323, WB-338) and Haplotype B (WB-403, WB-454, WB-459) extreme accessions are identified in the graph. Student's *t* test (*P*-value, 0.05). **C)** Bio14 value in extreme accessions belonging to haplotype A and B. **D)** Germination percentage and **E)** seedling area of extreme barley accessions analyzed after 4 d of flooding, followed by 5 days of recovery in soil using hulled seeds. Within each box, center lines denote median values, box limits extend from the 25th to the 75th percentile, whiskers denote 1.5 × interquartile range, and dots denote outliers. ANOVA followed by Tukey's HSD test (*P*-value < 0.05).

We performed an eGWAS to identify the genetic markers that were significantly associated with bio14 and bio18 variables. The linkage disequilibrium (LD) within the WHEALBI collection was estimated for the total 440 accessions in order to define robust genomic intervals and support the identification of putative candidate genes (Supplementary Table S2). The LD decay distance of the whole collection was estimated to be approximately 1.25 mb (*r*^2^ = 0.1) (Supplementary Fig. S2A). As expected, the frequency of recombination was lower in the centromeric region in all chromosomes (Supplementary Fig. S2B). Concerning the genome-wide associations, no significant signal was obtained for bio18 (Supplementary Fig. S3), while significant signals were found for bio14 (Fig. 3A, Supplementary Table S3).

On chromosome 1, a relatively narrow genomic region was defined using the LD decay distance and by considering the two significant marker-variable associations. Within this region, we identified gene models annotated as *dirigent protein DIR* (*HvDIR-like*) (*HORVU.MOREX.r2.1HG0000220*) and *paired amphipathic helix protein SIN3* (*HvSIN3-like*) (*HORVU.MOREX.r2.1HG0000280).* These genes are the only ones located within the consistent LD blocks of the two MTAs on chromosome 1 that also exhibited distinct haplotypes in barley accessions associated with extreme values of the bio14 variable. Local LD analyses were also carried out using the data of the 110 accessions, to get additional insights into the observed patterns of *HvDIR-like* and *HvSIN3-like*, showing that there is recombination between the two signals (Supplementary Fig. S4). Barley accessions at the extremes of the bio14 distribution, carry dissimilar alleles for both *HvDIR-like* and *HvSIN3-like*, identifying haplotype A and haplotype B. Accessions with intermediate bio14 values exhibit mixed alleles combinations. The statistical analysis, which includes FDR and Bonferroni correction (*α* = 0.05) corresponding thresholds of −log10(*P*) = 6.947, controlled for the presence of false positives, which were further minimized through the use of the BLINK multiple linear regression method. Although the number of georeferenced accessions available is limited, the combination of the analytical approaches used supports the reliability of the significant MTAs identified. Nonetheless, the availability of a larger number of georeferenced accessions might have enabled the detection of additional MTAs.

### Barley accessions from extreme precipitation regimes show differences in seed coat lignification patterns

Barley accessions characterized by extreme precipitation regimes during the driest month of the year (bio14) were associated with *HvDIR-like* gene haplotype A (WB-294, WB-323, WB-338) and B (WB-403, WB-454, WB-459) (Fig. 3, B and C, Supplementary Table S4). Both haplotype A and haplotype B accessions are classified as spring, six-row accessions, restricting the variability between the groups. While accessions belonging to haplotype A do not receive rain during the driest month of the year (0 mm of rain), accessions belonging to haplotype B receive around 50 mm of rain during this month (relatively low to moderate rainfall, depending on the context). In Arabidopsis, canonical *DIR* genes have been shown to correlate with increased lignification (Thamil et al., 2013), as together with LAC, they are essential to neolignan biosynthesis in seeds (Yonekura-Sakakibara et al., 2021). This finding is relevant to our previous results, which showed the involvement of a *HvLAC* gene in barley seed permeability and lignification (Gómez-Álvarez et al. 2023).

We analyzed the capacity of hulled seeds of barley accessions from extreme precipitation regimes to germinate in ground soil, thus mimicking a natural environment, after a flooding period of 4 d followed by 5 d of recovery. We observed that accessions that harbor haplotype A (which receives less water during the summer months) were characterized by better germination and seedling development during the recovery phase (40–80%) in comparison to accessions holding haplotype B (0–25%) (Fig. 3, D and E).

The barley annotated HvDIR-like protein lacks a canonical DIR domain but has a single jacalin-like lectin domain (JRL). In *Poaceae,* the family of dirigent proteins includes proteins holding not only a DIR domain but also a JRL domain (Esch and Schaffrath 2017; Esch et al. 2023). In the barley genome (MOREX, version 3), 76 genes are annotated as DIR proteins, holding a DIR domain only, a DIR and JRL domain, or a single or two JRL domains (Luo et al. 2022; Supplementary Fig. S5). The chimeric DIR–JRL group of proteins is not found in the *Arabidopsis thaliana* genome but is common in cereals (Esch et al. 2023).

**Figure 4.**
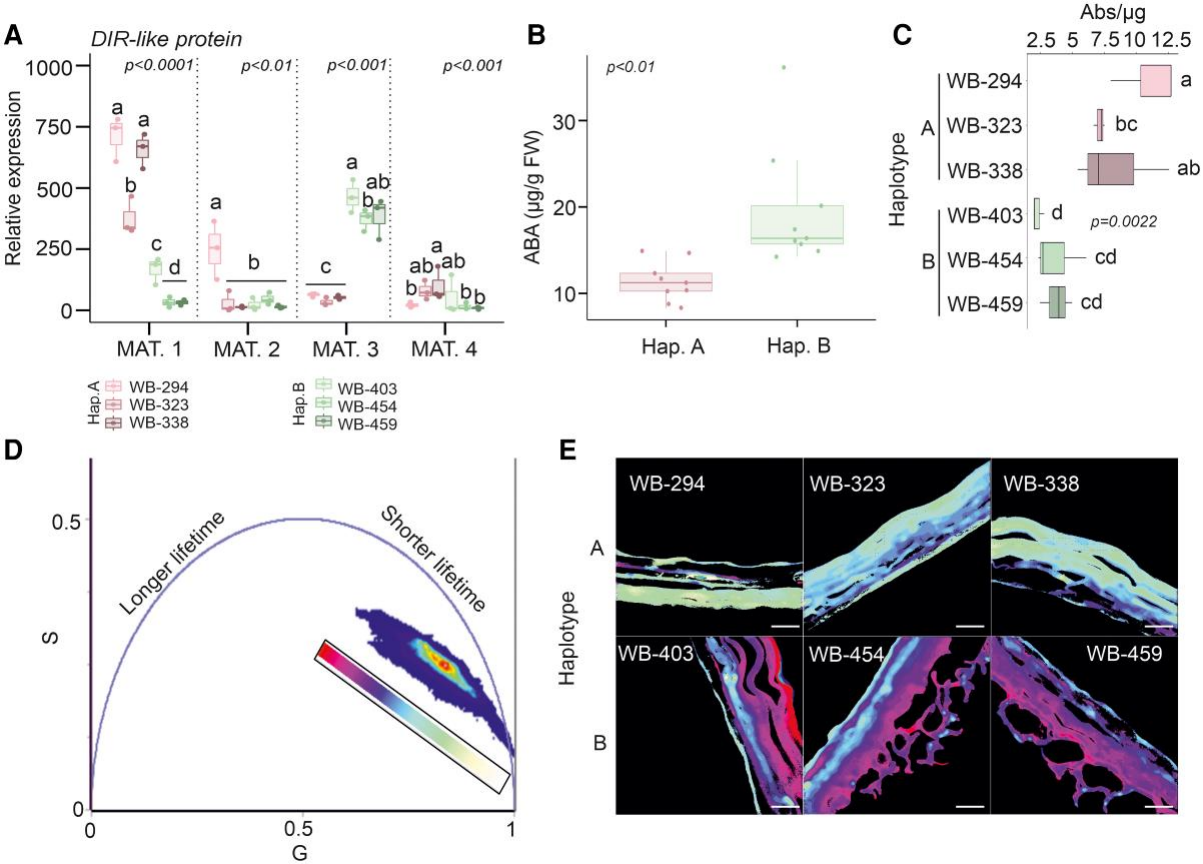
Gene expression, abscisic acid (ABA) quantification, seed permeability, and fluorescence lifetime imaging (FLIM) of extreme barley accessions. **A)** Gene expression analysis of the *HvDIR-like* gene during seed development in varieties holding haplotype A and B. **B)** ABA quantification in grain milk stage using whole seeds. Within each box, center lines denote median values; box limits extend from the 25th to the 75th percentile; whiskers denote 1.5 × interquartile range, and dots denote outliers. ANOVA followed by Tukey's HSD test (*P*-value < 0.05). **C)** Permeability analysis performed for bio14 extreme barley accessions, belonging to haplotype A and B. Within each box, center lines denote median values, box limits extend from the 25th to the 75th percentile, whiskers denote 1.5 × interquartile range, and dots denote outliers. ANOVA followed by Tukey's HSD test (*P*-value < 0.05). **D)** FLIM-derived Phasor plot showing the distribution of lifetimes measured, pixel-by-pixel, in a total of 55 images obtained from three accessions for each haplotype. A LUT is applied to associate a specific color to the position of each lifetime in the phasor plot. **E)** Color-coded image that indicates the lifetime of haplotypes. Scale bar 20 *μ*m.

Since the expression of Arabidopsis *DIR* genes has a known function during seed development for increased lignification (Yonekura-Sakakibara et al. 2021), we measured the barley *HvDIR-like* candidate expression at different stages after bolting. At seed maturation stage 3 (milk stage, Z75), the *HvDIR-like* gene showed an opposite trend in barley accessions holding extreme haplotypes in relation to bio14, with a higher expression in haplotype B (Fig. 4A). This suggests that accessions harboring haplotype B may be associated with an increased lignification, possibly influencing permeability of the seeds, thus subsequent germination. Accessions associated with haplotype A, on the other hand, may have an opposite phenotype.

Late seed maturation is facilitated by ABA, which is synthesized in the embryo and testa during seed development (Ali et al. 2021). In *Brassica* species, ABA has been shown to modulate the expression of *DIR-like* genes, which increase at the end of seed development (Paniagua et al. 2017). We thus measured ABA at barley seed maturation stage 3, observing that haplotype B accessions have an increased amount in comparison to haplotype A accessions (Fig. 4B). At this developmental stage, *HvDIR-like* expression is higher in haplotype B.

The different *HvDIR-like* genes were sequenced (Supplementary Fig. S6), and an amino acid shift from Lys (haplotype A) was identified in position 102 of the protein to Glu (haplotype B). In addition, a short deletion was observed in position −743 bp of the promoter of the accessions holding haplotype A. Due to the function of canonical DIR proteins in lignin and neolignan biosynthesis in Arabidopsis, we studied the permeability of the seeds in the two groups of accessions. The results showed that, as expected, a lower permeability of the seed was associated with haplotype B (Fig. 4C).

To study the lignification pattern, a further analysis was performed using fluorescence lifetime imaging microscopy (FLIM), in order to identify the fluorescence lifetime characteristics of the external seed surface. The measured lifetimes, upon phasor transformation (see Materials and Methods for details), showed an elongated distribution within the universal semi-circle, thus suggesting a complex mixture of autofluorescent components, putatively lignin, with heterogeneous characteristic lifetimes (Fig. 4D). The measured lifetimes were color-coded so that different compositions and/or abundances of autofluorescent components were revealed. In fact, haplotypes A and B were colored differently (Fig. 4E), which suggests that different lignins, in terms of composition and/or relative abundance of components, are present in their respective seed external layers.

Haplotype B appeared to be “red-shifted” with respect to haplotype A in the phasor plot, which suggests a higher relative abundance of components with longer characteristic lifetimes. Haplotype A was instead more enriched in components with shorter characteristic lifetimes. This suggests that, as expected, haplotypes A and B are associated with a dissimilar lignin pattern, with accessions harboring haplotype B being associated with a lower permeability of the seeds.

### Barley accessions from extreme precipitation regimes show dissimilar activation of dormancy after submergence

The haplotype of the *SIN3-like* gene, available on the LDs overlapping region of chr 1, is involved in dormancy. Haplotypes A and B of the *HvSIN3-like* gene are characterized by SNPs and amino acid variations (Fig. 3A). *HvSIN3-like* holds three paired amphipathic helix (PAH) domains and is similar to *SNL1* and *SNL2* from Arabidopsis (Supplementary Fig. S7), which are known to positively regulate primary dormancy (Wang et al. 2013). SNL1 and SNL2 are involved in histone deacetylation and can modulate the transcription of genes involved in the ABA pathway, a key hormone for dormancy regulation (Grzenda et al. 2009; Wang et al. 2013). In fact, ABA promotes seed dormancy and inhibits germination, while GA stimulates germination by mobilizing seed reserves. Under normal O_2_ conditions, a balance between ABA degradation and GA biosynthesis allows barley seeds to break dormancy and germinate. However, when seeds experience submergence stress, O_2_ availability becomes a limiting factor for GA synthesis, enhancing ABA's inhibitory effect on germination (Benech-Arnold et al. 2006). Indeed, little is known about the role of SNL1 and SNL2 in secondary dormancy, which can be activated as a consequence of stress, such as low O_2_ (Considine and Foyer 2023).

**Figure 5.**
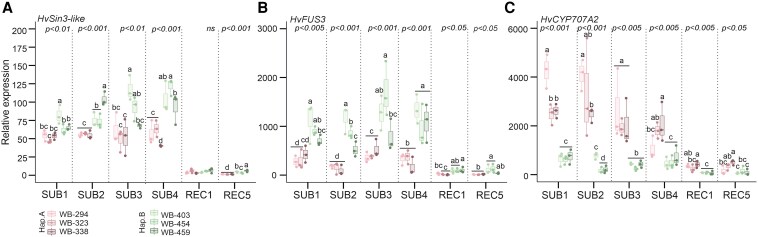
Gene expression during barley germination under submergence and recovery of extreme barley accessions. **A**) *HvSIN3-like,*  **B**) *HvFUS3-like* and **C**) *HvCYP707A2-like* gene expression during barley germination in embryos after 1, 2, 3, and 4 d of submergence and 1 and 5 d of recovery. Within each box, center lines denote median values, box limits extend from the 25th to the 75th percentile, whiskers denote 1.5 × interquartile range, and dots denote outliers. ANOVA followed by Tukey's HSD test (*P*-value <0.05).

**Figure 6.**
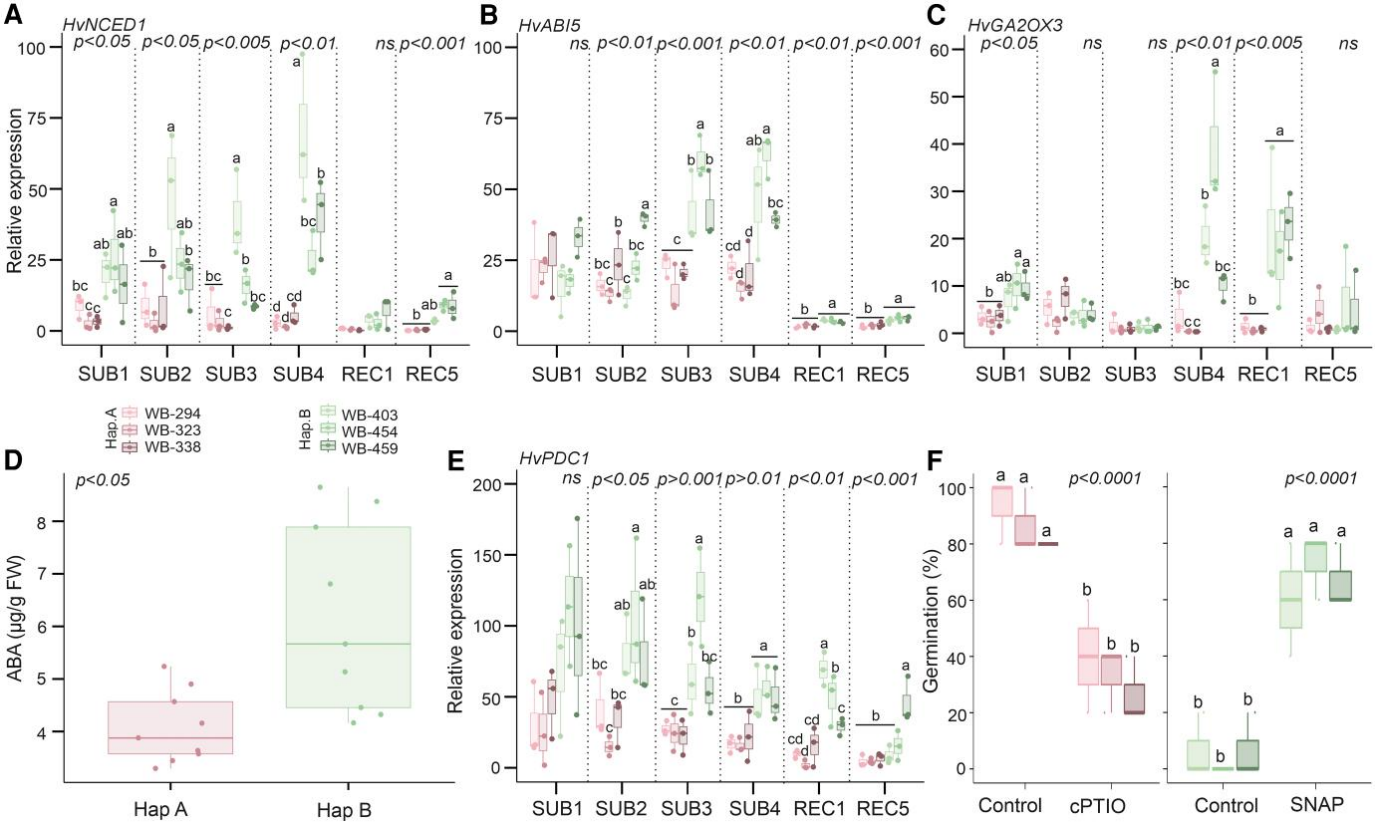
Dormancy and hypoxia-related molecular analysis during barley germination under submergence and recovery of extreme barley accessions. **A)**  *HvNCED1*, **B**) *HvABI5*, and **C**) *HvGA_2_OX_3_* gene expression analysis during barley germination in embryos after 1, 2, 3, and 4 d of submergence and 1 and 5 d of recovery. **D)** abscisic acid (ABA) quantification in haplotype A and haplotype B barley accessions during submergence at day 4. **E)**  *HvPDC1* gene expression analysis during barley germination in embryos after 1, 2, 3, and 4 d of submergence and 1 and 5 d of recovery. **F)** Germination percentage after 4 d of submergence followed by 5 d of recovery using nitric oxide (NO) scavenger (cPTIO, 0.5 mm) for haplotype A accessions, and NO donor (SNAP, 0.5 mm) for haplotype B accessions. Within each box, center lines denote median values, box limits extend from the 25th to the 75th percentile, whiskers denote 1.5 × interquartile range, and dots denote outliers. ANOVA followed by Tukey's HSD test (*P*-value < 0.05).

We measured *HvSIN3-like* expression in barley embryos excised from the grain during a time-course experiment in which grains were submerged for 1, 2, 3 and 4 d, followed by 1 and 5 d of recovery (Fig. 5A). During the four submergence days, a higher *HvSIN3-like* expression was observed in haplotype B accessions than in haplotype A, with an increasing trend. We also measured the expression of genes previously found to be regulated in the *snl1 snl2-1* Arabidopsis mutant and thus possible targets of *HvSIN3-like* (Wang et al. 2013). *HvFUSCA3* (*FUS3*), a seed master regulator that plays a critical role in seed dormancy through positive ABA regulation and GA negative regulation (Liu et al. 2023), was found to be upregulated in haplotype B (Fig. 5B). In contrast, *HvCYP707A2*, involved in ABA catabolism, was found to be downregulated in haplotype B (Fig. 5C). These results suggest that *HvSIN3-like* and its downstream targets are regulated differently across haplotypes. Given that *HvSIN3-like* is associated with the transcriptional regulation of dormancy in Arabidopsis (Wang et al. 2013), we measured the expression of ABA- (*HvNCED1* and *HvABI5*) and GA-related genes (*HvGA2OX3*), to assess whether haplotype B accessions were in a dormant state (Fig 6A, 6B, and 6C). We observed an induction of *HvNCED1* and *HvABI5* in haplotype B accessions, involved in ABA biosynthesis and signaling. Similar results were obtained with *HvGA2OX3*, which is involved in GA catabolism. This result shows that ABA and GA regulation correlate with the differential expression of *HvSIN3-like* and its targets. In addition, we measured the concentration of ABA at the end of submergence, showing a higher amount of ABA in haplotype B in comparison with haplotype A accessions (Fig. 6D). We thus measured the low O_2_-responsive genes *HvPDC1*, observing an induction in haplotype B accessions during submergence and recovery. Oxygen is a critical limiting factor for barley seed germination. The seed coverings influence O_2_ diffusion to the embryo, further shaping the germination response under hypoxic conditions (Bradford et al. 2008). These results support the hypothesis of secondary dormancy activation in haplotype B (Fig. 6E). Indeed, the application of NO donors, which are known to release dormancy, resulted in improved germination after soil flooding in barley accessions harboring haplotype B (Fig. 6F, Supplementary Fig. S8).

## Conclusions

Landraces and wild accessions from plant genetic resources can be studied in function of the climatic variables of their sites of origin. This approach allows the investigation of genomic regions associated with a particular environment. When studying the plant response to submergence, the rainfall regime of the original collection site is of interest, since a location characterized by strong precipitation during the germination phase of the crop may exert selection for adaptation traits.

In this work, we explored the bioclimatic data of wild accessions and landraces of the barley WHEALBI collection and found that bio14 and bio18 significantly correlated with the barley's capacity to germinate after submergence. Environmental GWAS of the bio14 variable along our wild and landrace barley subgroup identified an overlap between the LD regions of two significant marker-bioclimatic variable associations on chromosome 1. In this region, we identified a noncanonical *HvDIR-like*, a family which in Arabidopsis plays a role in lignin and neolignans biosynthesis, and a *HvSIN3-like*, which in Arabidopsis is involved in dormancy.

The barley panel harbors two haplotypes for the *HvSIN3-like* and *HvDIR-like* chromosomal regions in accessions at the extremes of the bio14 distribution. Haplotype A was characteristic of accessions originating from geographical regions with no rainfall during the driest months of the year and a higher permeability of the seed. On the other hand, haplotype B was characteristic of accessions originating from geographical regions with some precipitation during the driest months of the year and a lower seed permeability. Haplotypes A and B were also characterized by a dissimilar lignin pattern and germination capacity in soil after submergence, with haplotype B showing a reduced performance.

Our data collectively support the conclusion that barley accessions characteristic of regions with different amounts of rainfall during the driest months of the year have a dissimilar seed developmental program. This leads to a dissimilar composition and/or relative abundance of lignin on the seed surface, ultimately influencing seed permeability. This structural difference impacts seed germination when under prolonged flooding followed by recovery, activating or not a dormant state. Notably, seeds with a lower permeability become hypoxic, a condition that promotes secondary dormancy (Supplementary Fig. S9). Our data collectively confirm the hypothesis that seed maturation is a critical stage for barley response to subsequent submergence events occurring during seed germination. Moreover, they reveal genetic variation that supports a different germination behavior, shaped by rainfall regimes throughout the cultivation cycle.

## Materials and methods

### Selection of barley accessions, climatic, and agroecological characterisation of the panel

A sub-group of the WHEALBI barley collection (https://www.wheatinitiative.org/whealbi, last accession June 2025) that had been previously used for GWAS (Gómez-Álvarez et al. 2023) was explored, selecting 110 genotypes of wild relatives (*Hordeum spontaneum*) and landrace (*Hordeum vulgare*) accessions with available geographic data (Supplementary Table S1). The selection of these accessions was constrained by the availability of geographical coordinates at the original collection sites and was refined to avoid stratification; indeed, a geographical extent was defined spanning from longitude −20.26 (min) to 105.65 (max) and latitude 0.0 (min) to 75.58 (max). Genotypes derived from accessions outside this extent were excluded. All seeds used in this study originated from a 2020 batch, and only varieties exhibiting germination rates greater than 80% were selected.

The previously available data were used to define the STI: (% of germinated seeds after 2 d of submergence followed by 5 d of recovery/% of germinated seeds after 5 d in air) × 100 (Gómez-Álvarez et al. 2023). This index ranges from 1% to 100% and corresponds to the capacity to germinate well in the postsubmergence period in comparison to germination in air. When it is equal to 100%, the germination capacity in the recovery period after submergence is fully maintained. Unless otherwise stated, all the following environmental and genetic analyses were carried out in R (R Core Team 2021). Historical Bioclimatic precipitation data (1970 to 2000) were obtained from the WorldClim2 database (https://worldclim.org/, last accessed January 2024) using the R/*raster* package. The variables selected were: bio12, annual precipitation; bio13, precipitation of the wettest month; bio14, precipitation of the driest month; bio15, precipitation seasonality; bio16, precipitation of the wettest quarter; bio17, precipitation of the driest quarter; bio18, precipitation of the warmest quarter; bio19, precipitation of the coldest quarter. Correlation analyses among the variables and with the STI were performed using *R/cor*, while PCA was performed using *R/factoextra* (Kassambara and Mundt 2020)

The agroecological data of the barley accessions were extrapolated using the information available in the GAEZ v4 Data Portal of the Food and Agriculture Organization of the United Nations (FAO, https://gaez.fao.org/pages/data-viewer, last accessed in September 2023). The data viewer tool was explored, focusing on the land and water resources of the agroecological areas. For the cultivation calendar, data available from the passport of each accession were coupled with the information from the FAO GIEWS databases (http://www.fao.org/giews/en/, last accessed in September 2023).

### LD and eGWAS

The LD decay as a function of physical distance was calculated using genotyping data from the entire WHEALBI collection, including data on 440 genotypes derived from wild relatives, landraces, as well as commercial varieties. We thus randomly selected 12 K SNP markers from an initial set of approximately 100 K markers with a minimum allele frequency (MAF) of 0.1, using *–thin* function implemented in plink 1.9 (Purcell et al. 2007). The evolution of LD as a function of physical distance was evaluated considering all pairwise LD *r*^2^ estimates within each chromosome separately, with R/*Ldheatmap* (Shin et al. 2006), adapting the method used by Dell'Acqua and colleagues (Dell'Acqua et al., 2015; Woldeyohannes et al. 2022). To visualize trends of local LD in each chromosome, individual marker *r^2^* values were averaged, using 5 Mbps moving windows. LD decay was estimated as a function of physical distance, according to the Hill and Weir equation (Hill and Weir 1988). The LD decay distance was determined with a threshold of *r*^2^ equal to 0.1. Local LD analysis was visualized with Haploview software (Barrett et al. 2005).

The selected WHEALBI panel of 110 accessions was analyzed using exome sequencing (Bustos-Korts et al. 2019). The *GAPIT3* package in R was used to identify associations between the bioclimatic variables and the available SNPs, using a Bayesian information and linkage-disequilibrium iterative nested keyway (BLINK) (Huang et al. 2019) to consider population structure and LD to obtain the significant associations (Liu et al. 2016). LD windows were used to obtain the list of candidate genes from eGWAS. To ensure robustness, we applied statistical corrections for multiple testing. We used both False Discovery Rate (FDR) and Bonferroni corrections (*α* = 0.05). All associations identified using FDR were confirmed under this threshold. Additionally, the use of the BLINK algorithm—a refined version of FarmCPU—offered improved control over false positives and accounted for population structure more effectively than traditional GLM or MLM approaches (Liu et al. 2016).

### Phenotypic analysis, seed-coat permeability and NO donor and scavenger treatments

For the phenotypic analysis, plants were grown in Magenta GA-7 vessels (Merck, 77 × 77 × 97 mm) in a growth cabinet (Percival Scientific, Perry, IA, USA) at 20 °C and 50% RH in the dark. The lids of the Magenta GA-7 vessels used for air samples had a 2-cm diameter hole to allow O_2_ diffusion. Submerged seeds were treated for 5 d, adding 350 mL of demineralized water. After the submergence, seeds were transferred into pots containing a combination of soil and vermiculite for 5 d in control conditions. Phenotypic analysis of the seedlings was performed using the PhenoAIxpert Pro (LemnaTec).

The seed-coat permeability test was performed using the Yonekura-Sakakibara protocol (2021) with modifications for barley seeds, as previously described in Gómez-Álvarez et al. (2023).

Supplementation with NO donors and scavengers was performed as previously described for Gómez-Álvarez et al. (2023). Briefly, during submergence, haplotype A accessions were supplemented with the NO scavenger carboxy-PTIO potassium salt (cPTIO, 0.5 mm; Sigma–Aldrich), while haplotype B accessions were supplemented with the NO donor S-nitroso-N-acetylpenicillamine (SNAP, 0.5 mm; Sigma–Aldrich).

### DNA and RNA extraction, sequencing and gene expression analysis

Sequencing of the genomic material was performed using the Wizard Genomic DNA Purification Kit (Promega), according to the manufacturer's protocol. After the DNA extraction, PCR amplification was performed using the Phusion High Fidelity DNA Polymerase (ThermoFisher Scientific). The primers used are listed in Supplementary Table S5. Sequencing procedures are reported in Gómez-Álvarez et al. (2023).

For gene expression analysis during grain development, the barley accessions were grown at 20 °C under a photoperiod of 14 h light (120 *µ*mol^–2^ s^−1^). Sampling was performed as in Gómez-Álvarez et al. (2023), following the Zadoks scale for developmental stages: awn tipping (Z49; 1), spike above collar (Z55; 2), grain milk stage (Z75; 3), and grain dough stage (Z85; 4). Samples were collected uniformly across the spike to minimize variation in seed maturation. Experiments were performed using pools of five seeds for each barley accession. For gene expression during germination, seeds of barley accessions were submerged, and then the embryos were excised and collected at 1, 2, 3 and 4 d. After 4 d, the water was removed, and the seeds were maintained in the air for up to 4 d. Embryos were dissected at each time-point, pooled, frozen in liquid nitrogen, and stored at −80 °C until processing. For the recovery period sampling, the seminal root and the coleoptile were removed when present. Experiments were performed using pools of five seeds for each barley accession.

Embryo RNA extraction was performed using a Spectrum Plant Total RNA Kit (Sigma Aldrich) according to the manufacturer's protocol and as explained in Gómez-Álvarez et al. (2023). Two housekeeping genes were used for reference: *Tubulin1* and *Actin* (Hoang et al. 2013; Mendiondo et al. 2016). Relative expression levels were calculated using geNorm (https://genorm.cmgg.be/), with the tolerant accession WB-294 in air as the reference. Three biological replicates were used. Primers used are listed in Supplementary Table S5.

### Two-photon FLIM microscopy and data analysis

The autofluorescence intensity and the lifetime of barley seed cross-sections were acquired using an Olympus microscope coupled with a two-photon Ti:sapphire laser with 80-MHz repetition rate (MaiTai HP, SpectraPhysics) and a FLIMbox system (ISS, Urbana Champaign). The intrinsic fluorescence of the seed coat was excited at 760 nm and emissions were collected using a ×30 planApo silicon immersion objective (NA = 1.0) in the 380–570-nm range, where most lignin fluorescence is expected to fall (Donaldson 2013; Donaldson and Radotik 2013). For each measurement, a 512 × 512-pixel image (scan speed 10us/pixels) was collected. FLIM data were collected until a minimum of 10^5^ counts were obtained for each pixel of the region of interest. Calibration of the ISS Flimbox system was performed by measuring the known mono-exponential lifetime decay of Fluorescein at pH = 11.0 (i.e. 4.0 ns upon excitation at 760 nm, collection range: 380 to 570 nm). For the fluorescence lifetime imaging analysis, an image segmentation was performed by selecting only the seed coat region and the FLIM data analyzed using the phasor approach (Digman et al. 2008). In brief, for each image pixel, a fluorescence lifetime decay is measured, which is then converted by Fourier transform into a point, with coordinates *g* and *s*, in the phasor plot. Each pixel in the image thus corresponds to a point in the phasor plot and, *vice versa*, each point of the cluster in the phasor plot corresponds to an image pixel. Since each molecule has its own lifetime, by selecting different subregions of the cluster of points in the phasor plot, different species in the image can be highlighted with no *a priori* knowledge. FLIM data analysis was performed using SimFCS v. 4.0 software (Laboratory for Fluorescence Dynamics, University of California, Irvine).

### ABA quantification analysis

ABA levels were measured by using whole seeds. Quantification was carried out with an ELISA kit specific for ABA (AS20 4392, Agrisera, Sweden), following the manufacturer's protocol as in Gómez-Álvarez et al. (2023). For each barley accession, pools of five seeds for each sample were used in the analysis.

### Statistical analysis

Statistical analyses of the environmental data were performed using R 1.2.5019, (Bunn and Korpela 2008). The ggpubr package (https://rpkgs.datanovia.com/ggpubr/) was used to visualize the data and to compute the statistical analyses. Student's *t*-test was used to determine significant differences between pairs of means, and ANOVA followed by Fisher's LSD test was used to determine significant differences among means. The phylogenetic tree was performed by downloading all the protein sequences of *Hordeum vulgare Sin3-like* and *Arabidopsis thaliana SNL* genes available in the TAIR database (https://www.arabidopsis.org/, last accession in September 2023). Protein and phylogenetic tree alignment were performed with MEGA11: Molecular Evolutionary Genetics Analysis version 11 (Tamura et al. 2021).

### Accession numbers

Sequence data from this article can be found in the GenBank/EMBL data libraries under accession numbers *HORVU.MOREX.r2.1HG0000220* and *HORVU.MOREX.r2.1HG0000280*.

## Supplementary Material

kiaf563_Supplementary_Data

## Data Availability

Data are incorporated into the article and its online supplementary material. Further data are available on request.

## Supplementary data

The following materials are available in the online version of this article.

**Supplementary Table S1.** Characteristics of the wild and landraces barley accessions used for the genome wide association analysis.

**Supplementary Table S2.** Linkage disequilibrium (LD) calculation for each chromosome overall the Whealbi barley collection.

**Supplementary Table S3.** Genes located on the marker-bioclimatic variable association region on barley chromosomes.

**Supplementary Table S4.** Bioclimatic variables related to rainfall for the extreme barley accessions with haplotype A and B for bio14.

**Supplementary Table S5.** List of primers used for gene expression analysis.

**Supplementary Figure S1.** Correlation analysis (Spearman's correlation coefficient) for the monthly rainfall data for the original sites of the barley accessions.

**Supplementary Figure S2.** Linkage disequilibrium (LD) analysis on the Whealbi collection.

**Supplementary Figure S3.** Manhattan plot resulting from the GWAS analysis for bio18.

**Supplementary Figure S4.** Local linkage disequilibrium (LD) analysis of chromosome 1 related to the heritability study of *HvSin3-like* and *HvDIR-like*.

**Supplementary Figure S5.** Phylogenetic tree of all DIR-like proteins from barley with their domains.

**Supplementary Figure S6.** Alignment of DIR-like related sequences for haplotypes A and B.

**Supplementary Figure S7.** Phylogenetic tree for barley HvSin3-like and Arabidopsis SNL genes.

**Supplementary Figure S8.** Nitric oxide (NO) feeding experiment during the 4 d of submergence, followed by 5 d of recovery.

**Supplementary Figure S9.** Proposed model for the molecular mechanism involved in dissimilar germination after submergence of extreme barley accessions. Created with BioRender.com.

## Dive Curated Terms

The following phenotypic, genotypic, and functional terms are of significance to the work described in this paper:

SNL1 Gramene: AT3G01320
SNL1 Araport: AT3G01320

SNL2 Gramene: AT5G15020
SNL2 Araport: AT5G15020

FUS3 Gramene: AT3G26790
FUS3 Araport: AT3G26790

HvABI5 Gramene: HORVU.MOREX.r2.5HG0426550
HvABI5 Araport: HORVU.MOREX.r2.5HG0426550

HvActin Gramene: HORVU.MOREX.r2.1HG0001540
HvActin Araport: HORVU.MOREX.r2.1HG0001540

HvCYP707A2 Gramene: HORVU.MOREX.r2.2HG0086330
HvCYP707A2 Araport: HORVU.MOREX.r2.2HG0086330

HvDIR-like Gramene: HORVU.MOREX.r2.1HG0000220
HvDIR-like Araport: HORVU.MOREX.r2.1HG0000220

HvFUS3 Gramene: HORVU.MOREX.r2.3HG0236170
HvFUS3 Araport: HORVU.MOREX.r2.3HG0236170

HvGA2Ox3 Gramene: HORVU.MOREX.r2.1HG0019640
HvGA2Ox3 Araport: HORVU.MOREX.r2.1HG0019640

HvNCED1 Gramene: HORVU.MOREX.r2.4HG0280290
HvNCED1 Araport: HORVU.MOREX.r2.4HG0280290

HvPDC1 Gramene: HORVU.MOREX.r2.4HG0319840
HvPDC1 Araport: HORVU.MOREX.r2.4HG0319840

HvSIN3-like Gramene: HORVU.MOREX.r2.1HG0000280
HvSIN3-like Araport: HORVU.MOREX.r2.1HG0000280

HvTub1 Gramene: HORVU.MOREX.r2.1HG006725
HvTub1 Araport: HORVU.MOREX.r2.1HG006725
