# Supplementary material for: Rainfall patterns during barley seed development underlie genomic variation for germination after flooding
Source: Plant Physiol. 2025 Nov 9;199(3):kiaf563. doi: 10.1093/plphys/kiaf563 (PMC12637206; doi:10.1093/plphys/kiaf563)

Supplementary Data

Supplementary Table S2 – Linkage disequilibrium (LD) calculation for each chromosome overall the Whealbi barley collection.

Location	Mb
Chromosome 1	1.6838
Chromosome 2	1.3874
Chromosome 3	1.9497
Chromosome 4	2.6156
Chromosome 5	1.9994
Chromosome 6	1.1918
Chromosome 7	1.2933

Supplementary Table S3 – Genes located on the marker-bioclimatic variable association region on barley chromosomes 1 (A), 2 (B), and 3 (C). The gene selection was performed considering the linkage disequilibrium (LD) of each chromosome.

A)

Gene ID	Description
HORVU.MOREX.r2.1HG0000180	O-acyltransferase WSD1
HORVU.MOREX.r2.1HG0000190	Transposon Ty1-PR3 Gag-Pol polyprotein
HORVU.MOREX.r2.1HG0000200	Fatty acyl-CoA reductase
HORVU.MOREX.r2.1HG0000210	Albumin 1
HORVU.MOREX.r2.1HG0000220	Dirigent protein
HORVU.MOREX.r2.1HG0000230	disease resistance protein (TIR-NBS-LRR class)
HORVU.MOREX.r2.1HG0000240	Leucine-rich repeat receptor-like protein kinase family protein
HORVU.MOREX.r2.1HG0000250	Defensin-like (DEFL) family protein
HORVU.MOREX.r2.1HG0000260	disease resistance protein (TIR-NBS-LRR class)
HORVU.MOREX.r2.1HG0000270	Disease resistance protein (TIR-NBS-LRR class) family
HORVU.MOREX.r2.1HG0000280	Paired amphipathic helix protein Sin3
HORVU.MOREX.r2.1HG0000290	Pm3-like disease resistance protein
HORVU.MOREX.r2.1HG0000300	Pm3-like disease resistance protein
HORVU.MOREX.r2.1HG0000310	Pm3-like disease resistance protein
HORVU.MOREX.r2.1HG0000320	Pm3-like disease resistance protein
HORVU.MOREX.r2.1HG0000330	Phosphatidylinositol 3-kinase
HORVU.MOREX.r2.1HG0000340	Retrovirus-related Pol polyprotein from transposon TNT 1-94
HORVU.MOREX.r2.1HG0000350	Retrovirus-related Pol polyprotein from transposon TNT 1-94
HORVU.MOREX.r2.1HG0000360	NADH pyrophosphatase
HORVU.MOREX.r2.1HG0000370	Retrovirus-related Pol polyprotein from transposon TNT 1-94
HORVU.MOREX.r2.1HG0000380	Retrotransposon protein, putative, unclassified
HORVU.MOREX.r2.1HG0000390	Glucan 1,3-beta-glucosidase
HORVU.MOREX.r2.1HG0000400	SWAP (Suppressor-of-White-APricot)/surp -containing protein

B)

Gene ID	Description
HORVU.MOREX.r2.2HG0103500	Inter-alpha-trypsin inhibitor heavy chain-like protein
HORVU.MOREX.r2.2HG0103510	Proteasome subunit alpha type
HORVU.MOREX.r2.2HG0103520	Adipocyte plasma membrane-associated protein
HORVU.MOREX.r2.2HG0103530	DUF1677 family protein (DUF1677)
HORVU.MOREX.r2.2HG0103540	Retrotransposon protein, putative, unclassified
HORVU.MOREX.r2.2HG0103550	ARM repeat superfamily protein
HORVU.MOREX.r2.2HG0103560	NADH-quinone oxidoreductase subunit B
HORVU.MOREX.r2.2HG0103570	Retrovirus-related Pol polyprotein from transposon TNT 1-94
HORVU.MOREX.r2.2HG0103580	Retrovirus-related Pol polyprotein from transposon TNT 1-94
HORVU.MOREX.r2.2HG0103590	DWNN domain, a CCHC-type zinc finger-like protein
HORVU.MOREX.r2.2HG0103600	Zf-RanBP domain-containing protein
HORVU.MOREX.r2.2HG0103610	Retrovirus-related Pol polyprotein from transposon TNT 1-94
HORVU.MOREX.r2.2HG0103620	Retrovirus-related Pol polyprotein from transposon TNT 1-94
HORVU.MOREX.r2.2HG0103630	Retrovirus-related Pol polyprotein from transposon TNT 1-94
HORVU.MOREX.r2.2HG0103640	Retrovirus-related Pol polyprotein from transposon TNT 1-94
HORVU.MOREX.r2.2HG0103650	ATP binding microtubule motor family protein

HORVU.MOREX.r2.2HG0103660	Zinc finger MYM-type-like protein
HORVU.MOREX.r2.2HG0103670	Zinc finger MYM-type protein 1
HORVU.MOREX.r2.2HG0103680	Retrovirus-related Pol polyprotein from transposon TNT 1-94
HORVU.MOREX.r2.2HG0103690	Protein FAR1-RELATED SEQUENCE 5
HORVU.MOREX.r2.2HG0103700	Protein FAR1-RELATED SEQUENCE 5
HORVU.MOREX.r2.2HG0103710	Tyrosine recombinase XerC
HORVU.MOREX.r2.2HG0103720	F-box family protein
HORVU.MOREX.r2.2HG0103730	Replication protein A 32 kDa subunit
HORVU.MOREX.r2.2HG0103740	ATP synthase subunit b
HORVU.MOREX.r2.2HG0103750	Transducin/WD40 repeat-like superfamily protein
HORVU.MOREX.r2.2HG0103760	UPF0115 protein YfcN
HORVU.MOREX.r2.2HG0103770	Kinase family protein
HORVU.MOREX.r2.2HG0103780	Exportin-T
HORVU.MOREX.r2.2HG0103790	50S ribosomal protein L15
HORVU.MOREX.r2.2HG0103800	Rela/spot homolog 3 family protein
HORVU.MOREX.r2.2HG0103810	Transcription initiation factor TFIID subunit 9
HORVU.MOREX.r2.2HG0103820	Transmembrane protein 53
HORVU.MOREX.r2.2HG0103830	Alpha/beta-hydrolase superfamily protein
HORVU.MOREX.r2.2HG0103840	DNA-directed RNA polymerase III subunit

C)

Gene ID	Description
HORVU.MOREX.r2.3HG0228680	Callose synthase-like protein
HORVU.MOREX.r2.3HG0228690	Glycerol-3-phosphate acyltransferase
HORVU.MOREX.r2.3HG0228700	GTP-binding protein
HORVU.MOREX.r2.3HG0228710	GDSL esterase/lipase
HORVU.MOREX.r2.3HG0228720	RING/FYVE/PHD zinc finger superfamily protein
HORVU.MOREX.r2.3HG0228730	Serine/threonine protein phosphatase 7 long form isogeny
HORVU.MOREX.r2.3HG0228740	Pentatricopeptide repeat-containing protein
HORVU.MOREX.r2.3HG0228750	Phospholipase A1
HORVU.MOREX.r2.3HG0228760	Phospholipase A1
HORVU.MOREX.r2.3HG0228770	GDSL esterase/lipase
HORVU.MOREX.r2.3HG0228780	Retrovirus-related Pol polyprotein from transposon TNT 1-94
HORVU.MOREX.r2.3HG0228790	MBOAT (membrane bound O-acyl transferase) family protein
HORVU.MOREX.r2.3HG0228800	Transposon protein, putative, Pong sub-class, expressed
HORVU.MOREX.r2.3HG0228810	F-box/RNI-like superfamily protein
HORVU.MOREX.r2.3HG0228820	F-box family protein
HORVU.MOREX.r2.3HG0228830	alpha/beta-Hydrolases superfamily protein
HORVU.MOREX.r2.3HG0228840	Phospholipase A1
HORVU.MOREX.r2.3HG0228850	Transposon protein, putative, Pong sub-class

Supplementary Table S4 – Bioclimatic variables related to rainfall for the extreme barley accessions with haplotype A and B for bio14.

Varieties	bio12	bio13	bio14	bio15	bio16	bio17	bio18	bio19
**WB-294**	268	62	0	98	158	0	0	158
**WB-323**	105	20	0	94	59	0	0	59
**WB-338**	107	34	0	104	68	8	11	48
**WB-403**	1061	98	73	8	282	249	259	261
**WB-454**	796	105	46	26	274	142	149	260
**WB-459**	877	104	49	21	280	158	158	243

Supplementary Table S5 – List of primers used for gene expression analysis.

Name	Gene ID	Forward primer	Reverse primer	References
HvABI5	HQ456390.1 HORVU.MOREX.r2.5HG0426550	CCGGTCCCTGTTGCCCCTAAAG	CGCCGCCCATACCGAG	Collin et al., 2020
HvActin	AY145451, HORVU.MOREX.r2.1HG0001540	CCAAAAGCCAACAGAGAGAA	GCTGACACCATCACCAGAG	Hoang et al., 2013
HvCYP707A2	HORVU.MOREX.r2.2HG0086330	CTGAGCAACAATGGAGGCC	TTATCCACCCATCCACCCCT	
HvDIR-like	HORVU.MOREX.r2.1HG0000220	ATGGTATGTGTTTGATCCTCT	TCATGGAGTAATGTAGAGCC	
HvFUS3	HORVU.MOREX.r2.3HG0236170	AATCCCATCACCAGCTGACA	TGACTTGCCAGGTTAGCTGA	
HvGA2ox3	24223269 HORVU.MOREX.r2.1HG0019640	GAGAGCAGAGCCTGTACAAG	TGGCTACCTGTGGAAGTGAG	Hoang et al., 2013
HvNCED1	HORVU.MOREX.r2.4HG0280290	CCAGCACTAATCGATTCC	GAGAGTGGTGATGAGTAA	
HvPDC1	HORVU.MOREX.r2.4HG0319840	CAGGATGAGCAACAAGATGG	GTTCAGGAACTCGACGGTTG	
HvSIN3-like	HORVU.MOREX.r2.1HG0000280	CCGGAAGATCATGAGGACGA	TGCTTGGCGAGTGGTTTAAC	
HvTub1	AK250165.1, HORVU.MOREX.r2.1HG0067250	CAGGCTTGTTTCTCAGGTCA	ATTCAGAGCACCGTCAAACC	Mendiondo et al., 2015

Supplementary Figure S1 – Correlation analysis (Spearman's correlation coefficient) for the monthly rainfall data for the original sites of the barley accessions.

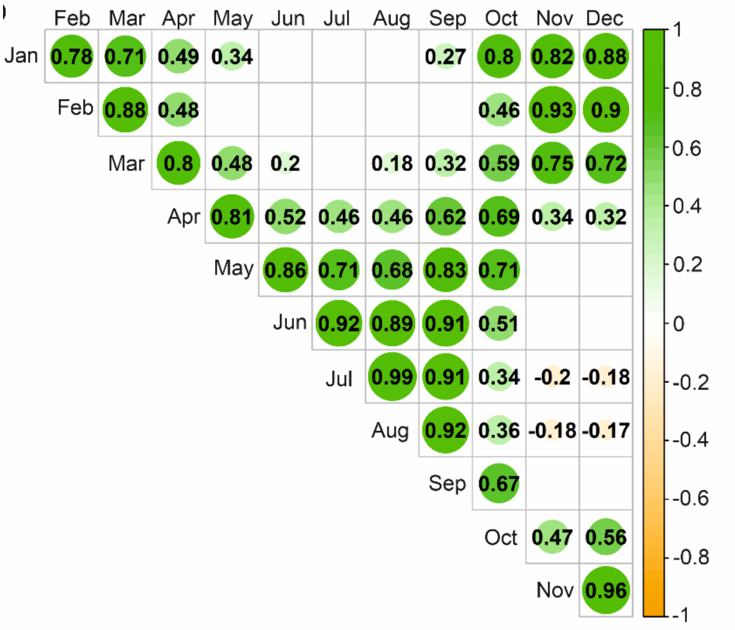

Supplementary Figure S2 – Linkage disequilibrium (LD) analysis on the Whealbi collection. **A)** Linkage disequilibrium (LD) decay. **B)** LD of the Whealbi collection separated by chromosomes, in which each line with a different colour represents a different chromosome. The triangle represents the position of the centromere.

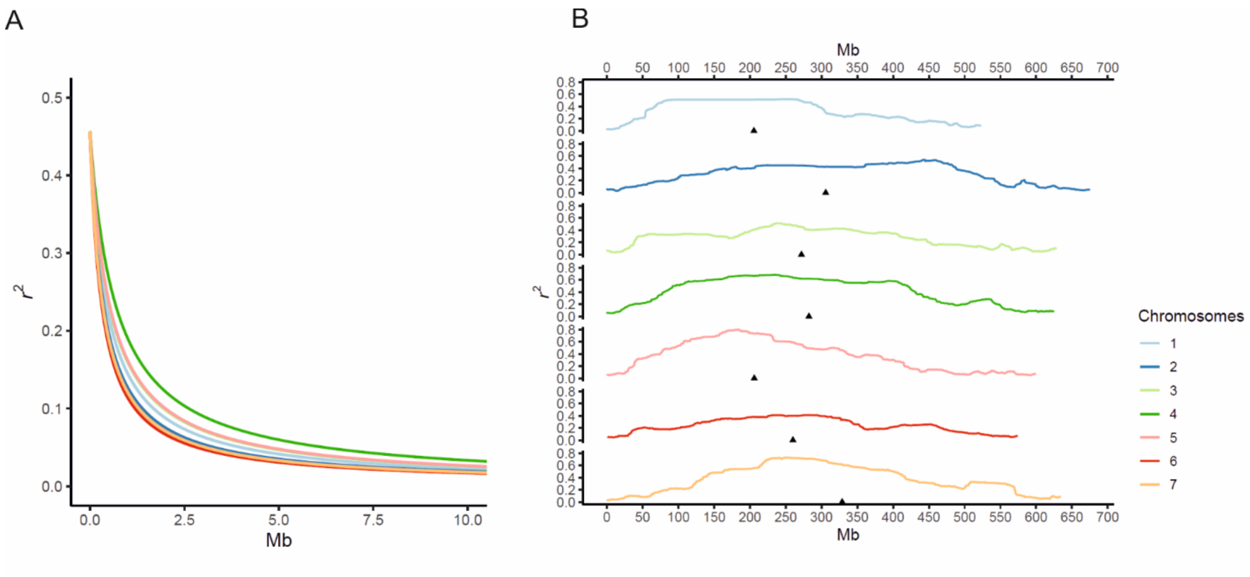

Supplementary Figure S3 – Manhattan plot resulting from the GWAS analysis for bio18.

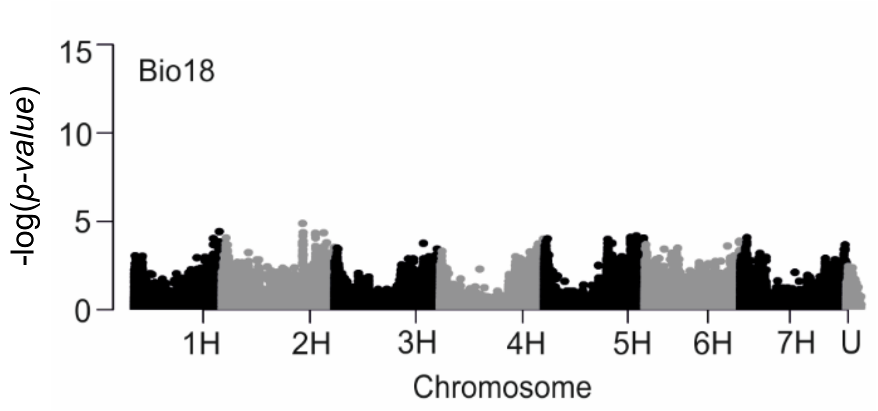

Supplementary Figure S4 – Local linkage disequilibrium (LD) analysis of chromosome 1 related to the heritability study of *HvSin3-like* and *HvDIR-like*.

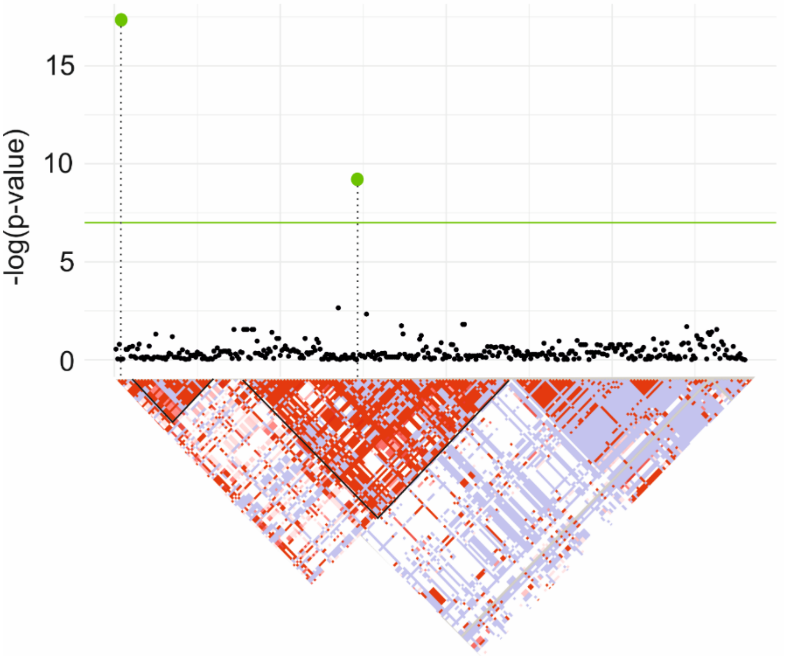

Supplementary Figure S5 – Phylogenetic tree of all DIR-like proteins from barley with their domains. Outgroup is a DIR-like gene from *Musa acuminata*. The scale bar represents the proportion of amino acid differences between sequences.

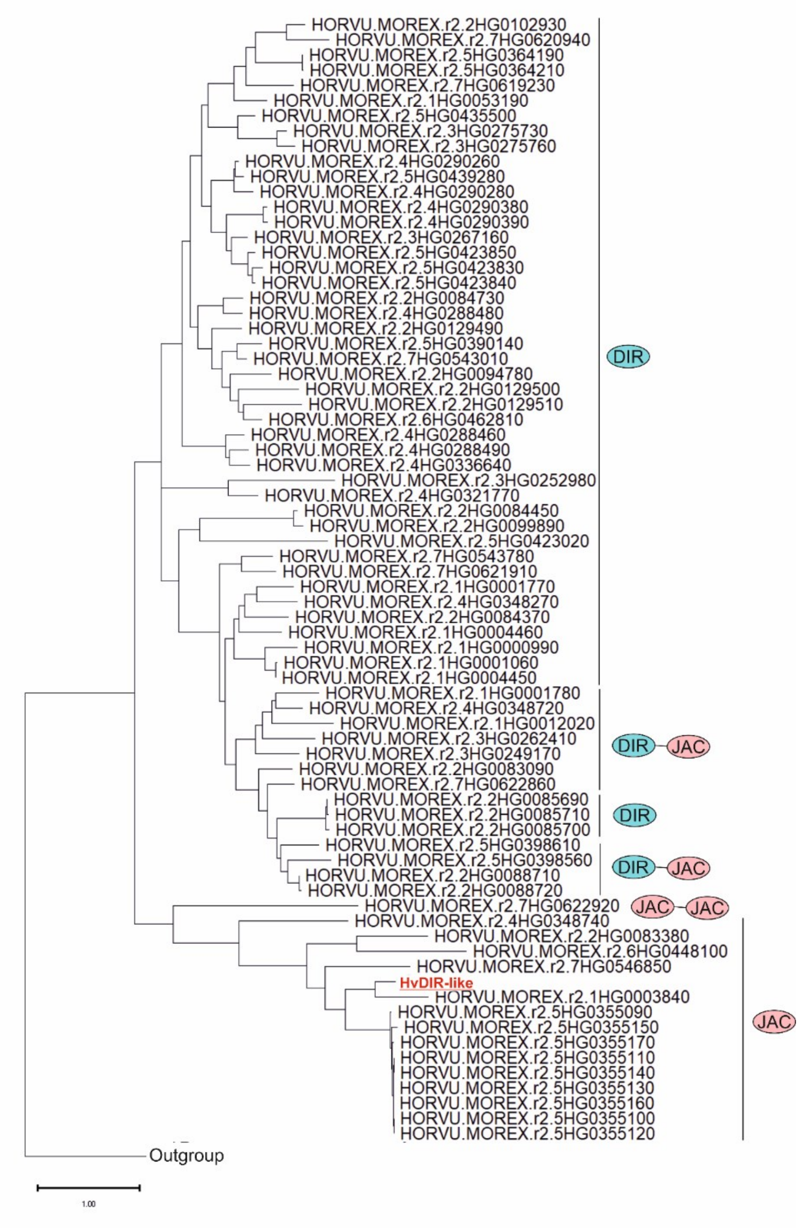

Supplementary Figure S6 - Alignment of DIR-like related sequences for haplotypes A and B. **A)** Protein sequence and **B)** promoter region of the *HvDIR-like* gene.

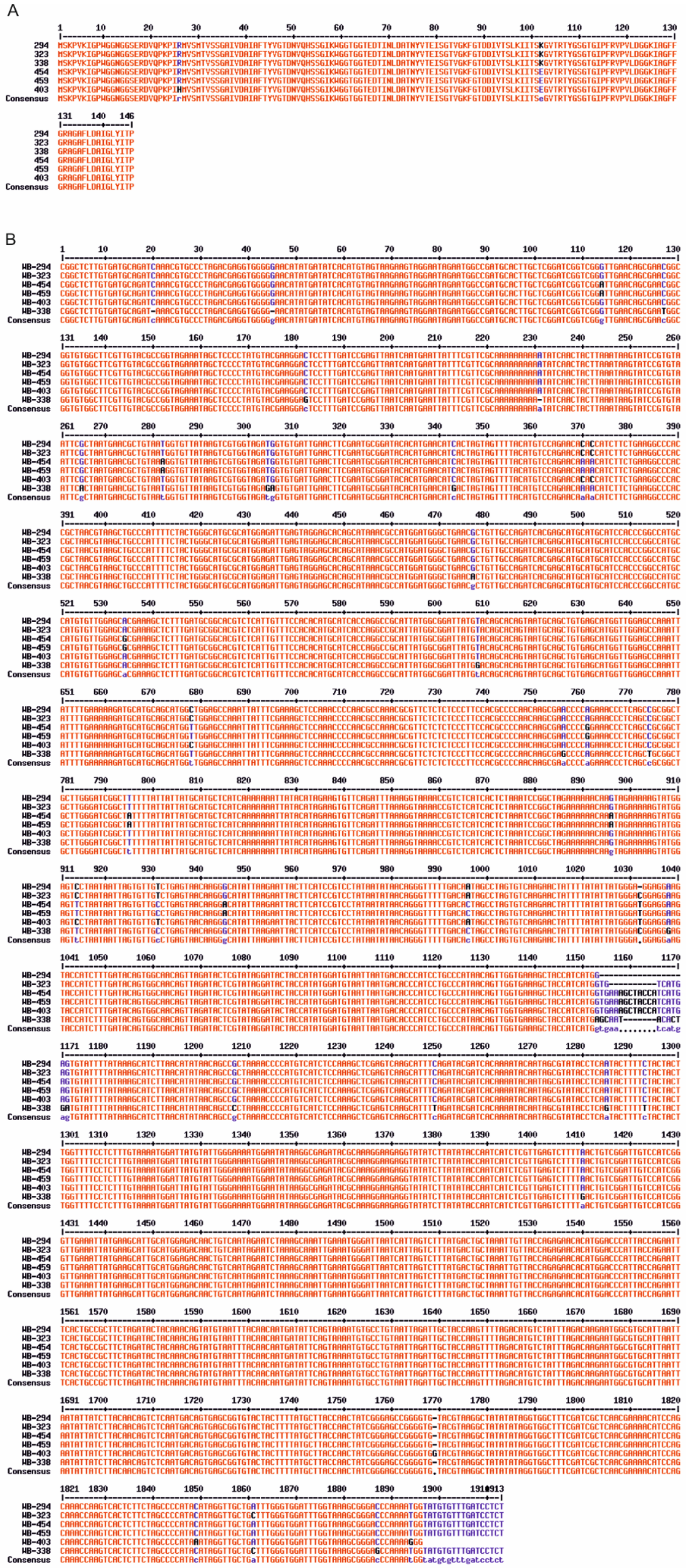

Supplementary Figure S7 - Phylogenetic tree for barley HvSin3-like and Arabidopsis SNL proteins. Outgroup is a Sin3 protein from *Musa acuminata.* The scale bar represents the proportion of amino acid differences between sequences.

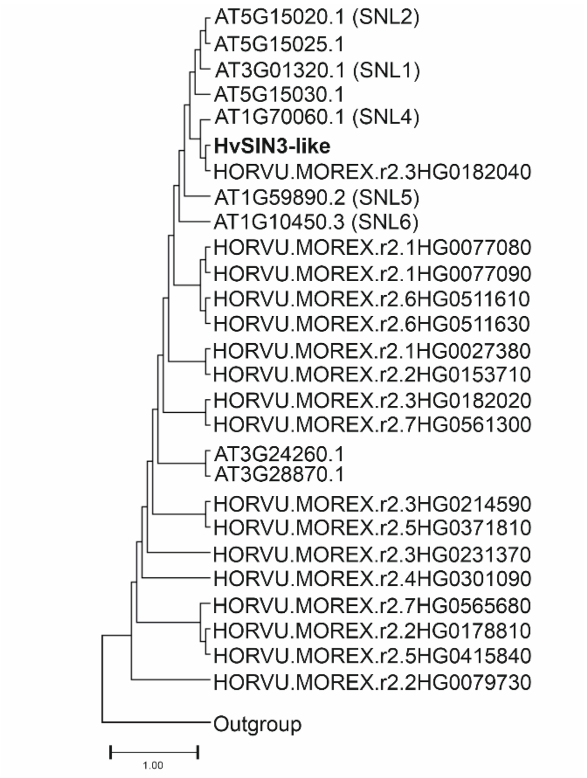

Supplementary Figure S8 – Nitric oxide (NO) feeding experiment during the 4 days of submergence followed by 5 days of recovery. NO scavenger (cPTIO, 0.5 mM) for haplotype B accessions, and NO donor (SNAP, 0.5 mM) for haplotype A accessions were used. Within each box, center lines denote median values, boxes limits extend from the 25th to the 75th percentile, whiskers denote 1.5x interquartile range and dots denote outliers. ANOVA followed by Tukey's HSD test (p-value <0.05).

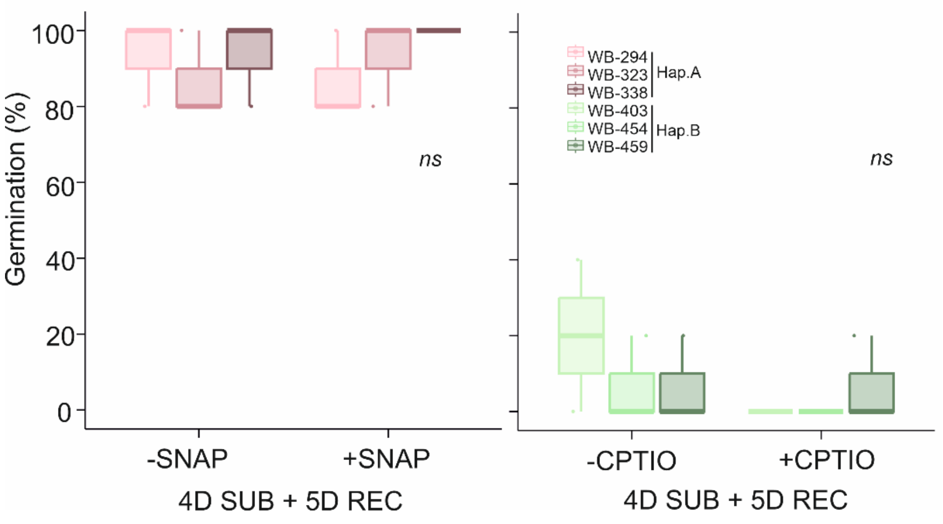

Supplementary Figure S9 - Proposed model for the molecular mechanism involved in dissimilar germination after submergence of extreme barley accessions.

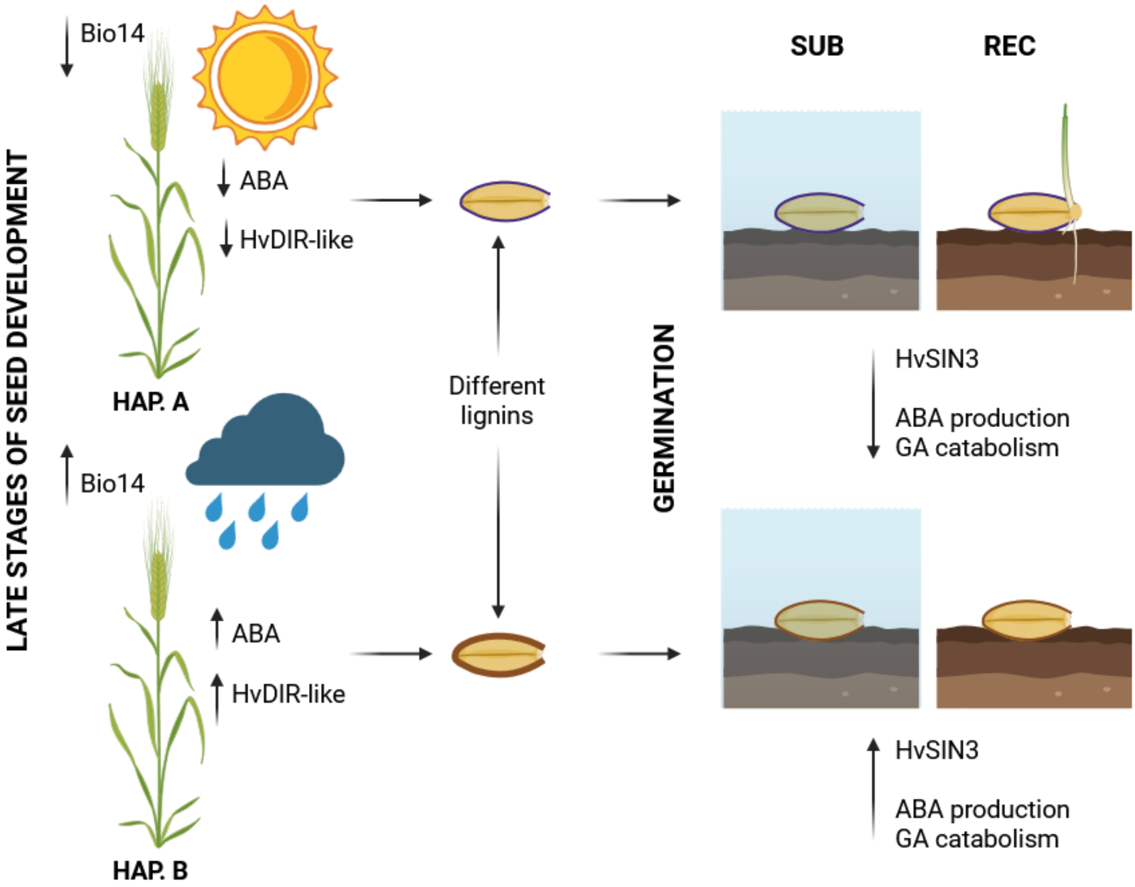

Supplement: kiaf563_Supplementary_Data [file kiaf563_supplementary_data.zip › Supplementary_301025.pdf]
